# Associations Between Personal Views of Aging and Quality of Life in Midlife and Older Age: The Mediating Role of Psychological Resilience

**DOI:** 10.3390/healthcare13222906

**Published:** 2025-11-14

**Authors:** Enrico Sella, Elena Carbone, Erika Borella

**Affiliations:** Department of General Psychology, University of Padova, 35131 Padova, Italy; elena.carbone@unipd.it (E.C.); erika.borella@unipd.it (E.B.)

**Keywords:** aging, awareness of age-related change, felt age, psychological resilience, quality of life

## Abstract

**Background/Objectives**: Ensuring quality of life (QoL) is a key aspect of promoting healthy aging. This cross-sectional study investigated whether and to what extent personal views of aging (VoA)—individuals’ perceptions, attitudes, and expectations regarding their own aging—and psychological resilience are associated with QoL and its domains in middle-aged and older adults. **Methods**: A sample of 224 individuals (46–85 years) was recruited. All participants reported their felt age (FA) and completed the Awareness of Age-Related Change (AARC) questionnaire, assessing awareness of age-related gains (AARC-Gains) and losses (AARC-Losses). They also completed the World Health Organization Quality of Life assessment (WHOQOL-BREF) and the Connor–Davidson Resilience Scale for psychological resilience. Multiple regressions and path analyses were run to examine the associations among personal VoA, psychological resilience, and QoL. **Results**: Regression analyses showed that AARC-Gains and AARC-Losses (but not FA) predicted overall QoL, with AARC-Losses and, to some extent, FA also explaining specific QoL domains. Resilience also emerged as a significant positive predictor for overall QoL and its psychological and environmental domains. Path analyses confirmed and extended the role of personal VoA and resilience on QoL. Resilience directly influenced QoL and its domains, in turn mediating the effects of personal VoA, depending on the specific facets of VoA and the QoL domains examined. **Conclusions**: These findings suggest that promoting positive/correct personal views of aging and fostering psychological resilience may be promising healthcare strategies for enhancing QoL in adulthood into older age.

## 1. Introduction

Subjective views of aging (VoA) is an umbrella term referring to individuals’ beliefs, experiences, and evaluations of older adults as a social group, in general, and their own aging process [1]. Interest in the construct of VoA and experimental studies on it have constantly increased in recent decades [1]. These views have been shown to influence a range of health outcomes in aging, including physical and mental health as well as longevity [2,3]. They have also been recognized as self-regulatory mechanisms that influence developmental outcomes across adulthood and older age [4]. Despite these promising findings, it remains unclear how VoA relate to other relevant dimensions of aging, such as quality of life (QoL). Identifying factors that promote QoL in older age represents a key priority, especially considering the increase in life expectancy (e.g., [5,6]) and the need to focus on modifiable risk factors (e.g., [7]), among which VoA may, thus, play a crucial role.

QoL is a subjective, multidimensional evaluation of one’s life circumstances in relation to personal goals, expectations, standards, and concerns [8,9] that changes across the lifespan in response to biological, psychological, and social factors, often following nonlinear and unpredictable trajectories (e.g., [10,11]). It is, therefore, considered a key multidimensional outcome for health and well-being in the successful and active aging field [12]. However, research on factors influencing QoL in middle-aged and older adults has mainly focused on physiological and behavioral risk factors for older adults’ health and QoL (e.g., [13]), such as the incidence of neurocognitive diseases (e.g., [7,14]). Far less attention has been given to the subjective experience of aging, as reconceptualized within the VoA framework [1], and its potential association with QoL in midlife and older adulthood. A recent meta-analysis [15] provides initial evidence supporting the impact of generalized VoA—representing broader beliefs about aging or older adults as a social category—on QoL: more general positive attitudes toward aging have been shown to be associated with better QoL (measured using overall scores from the WHOQOL assessment), whereas more negative ones predict poorer QoL.

However, VoA can also be assessed by examining personal VoA, that is individuals’ beliefs and expectations regarding their own aging process. A widely used unidimensional indicator of personal VoA is subjective age, or felt age (FA), reflecting how old individuals feel compared to their chronological age. It is considered a global indicator of self-perceptions of aging and thus a central construct of the personal aging experience [16]. FA, for instance, represents a powerful antecedent and predictor of health-related developmental and successful aging outcomes over the adult life course [17]. A recent meta-analysis by Westerhof et al. [3] also indicates that a youthful FA is associated with better health-related outcomes closely related to QoL, such as perceived mental and physical health, subjective well-being, and life satisfaction. In contrast, chronological age, which is the time metric most frequently used to indicate individuals’ standing in the life span, fails to fully capture the complexity of the aging process, as it excludes the personal experience of it [17] and its influence on various psychological outcomes (e.g., [3]).

More recently, the shift from a uni- to a multidimensional approach to conceptualize personal VoA has given rise to the Awareness of Age-Related Change (AARC; [18]) construct, which distinguishes between perceived age-related gains (AARC-Gains) and losses (AARC-Losses) across various domains of functioning, such as health, cognition, social relationships, and lifestyle, allowing new facets of personal VoA to emerge. AARC-Gains and AARC-Losses have been shown to be positively and negatively linked, respectively, with better health-related outcomes in terms of physical functioning, well-being, and life satisfaction [4,19,20].

Notwithstanding the plethora of evidence of meaningful associations between personal VoA and health-related outcomes closely related to QoL (e.g., [4,19,20]), how personal VoA relate to QoL remains not well understood. Such an association has not been examined at all in connection to the distinct domains of QoL (physical and psychological health, social relationships, and environment).

There is now, indeed, growing interest in identifying the potential (psychological) pathways linking VoA to QoL (and its domains). Among psychological factors [3,21], psychological resilience—defined as the personal ability to cope with and overcome/adapt to adversities while maintaining or regaining psychological well-being in the face of hardship [22]—is recognized as a key predictor of successful and active aging and QoL in older adulthood [12]. In midlife and older age, psychological resilience has emerged as a major protective factor, with older adults reporting higher psychological resilience also displaying better outcomes closely related to QoL, such as higher physical and mental health, greater life satisfaction than younger adults, and increased longevity [23,24]. Such a pattern of results is confirmed by a recent meta-analysis [23] among community-dwelling middle-aged and older adults, which found moderate positive associations between psychological resilience and multiple health indicators (e.g., lower depressive symptoms, higher self-rated health), as well as contextual resources, such as higher education and social support. Interestingly, some suggestions have been made regarding the role of psychological resilience in mediating the impact of VoA on various psychological outcomes. Ribeiro-Gonçalves et al. [25] found, among older adults, that psychological resilience mediated the effect of VoA (assessed as perceived ageism) on psychological distress and loneliness. Chen et al. [26] reported that, in community-dwelling middle-aged and older adults, psychological resilience mediated the association between personal VoA—measured as self-perceptions of aging related to ongoing personal development (AgeCog)—and subjective well-being, during the COVID-19 pandemic.

Despite promising findings suggesting that psychological resilience may influence distinct health-related and contextual aspects closely related to QoL, evidence on the associations between resilience and QoL in its multidimensionality (overall and across its four distinct domains) in middle-aged and older adults is still lacking. Of more interest, evidence linking different personal VoA facets and overall QoL, as well as its domains, through psychological resilience has yet to be further examined. A closer understanding of the psychological pathways linking personal VoA and QoL (both overall and domain-specific), such as psychological resilience, which appear to play a mediating role in various health outcomes (e.g., [23,24,25,26]), may help identify the antecedents and correlates of QoL in midlife and older age. Moreover, previous evidence has shown that age-related changes in health domains closely related to QoL (e.g., physical and mental functioning) may emerge among middle-aged adults and continue into older age (e.g., [27,28]). Psychological resilience, in turn, becomes paramount for adapting to age-related challenges and transitions that begin in midlife (e.g., [29,30]).

The aims were to examine the associations among personal VoA facets (i.e., FA and AARC-Gains and AARC-Losses), psychological resilience, and QoL among community-dwelling middle-aged and older adults. We also tested whether the associations between personal VoA and QoL are mediated by resilience as a psychological factor. Towards these aims, we measured QoL, not only globally but also considering its main domains (physical and psychological health, social relationships, and environment), using the WHOQOL-BREF [8], which provides both overall and domain-specific QoL assessments. Personal VoA facets were assessed with FA through a single-item question (e.g., [31]), as classically performed, and the AARC 50-item questionnaire (AARC-50; [32]), capturing both AARC-Gains and AARC-Losses in various domains of functioning; psychological resilience was measured with the 10-item Connor–Davidson Resilience Scale (CD-RISC-10; [33]). These associations were therefore assessed from midlife, as evidence on personal VoA (including FA and AARC) indicates that midlife is a life phase during which self-reflections about one’s age and aging begin and continue to intensify into older adulthood (see [34,35,36,37]).

In line with previous evidence (e.g., [3,15]), we expected a youthful FA to be associated with better overall QoL and its health-related domains (physical and psychological), as well as with the social and environmental domains, which are explored here for the first time. Consistent with previous evidence linking AARC dimensions to various health-related aspects of QoL in aging (e.g., [4]), we expected that higher AARC-Gains would be associated with higher QoL, and higher AARC-Losses with poorer QoL [4,19,20], across overall QoL and all QoL domains.

Because psychological resilience has been shown to be associated with some aspects related to QoL with aging and among older adults (e.g., [38,39]), we expected higher levels of resilience to be positively associated with better overall QoL. In line with previous evidence on psychological resilience and health in aging populations (e.g., [23,24]), we also expected this positive association to extend to the physical and psychological QoL domains. The associations between resilience and the underexamined social relationships and environment domains of QoL were also explored, where we expected positive associations.

Based on growing evidence from the VoA literature (e.g., [1,25,26]) and the recognized role of resilience as a psychological resource supporting adaptation in aging (e.g., [12,40]), we also tested a series of mediation models including personal VoA (FA, AARC-Gains, and AARC-Losses), psychological resilience, and QoL (overall and by domain). Based on these premises, we expected personal VoA to directly affect QoL. We further expected resilience to positively mediate the associations between personal VoA (e.g., youthful FA, higher AARC-Gains, lower AARC-Losses) and QoL, including, exploratorily, all QoL domains. If supported, the effect of personal VoA would thus be both direct and indirect, mediated by resilience.

In our models, chronological age, gender, and education were included as covariates, given their established relevance in shaping health and QoL outcomes [11,41].

## 2. Materials and Methods

### 2.1. Participants

This cross-sectional study involved Italian community-dwelling volunteer participants who were recruited through word-of-mouth dissemination, informal networks, and acquaintances [42]. Inclusion criteria included the following: (i) age range between 45 and 85 years, (ii) no history or diagnosis of physical or mental health issues, assessed through a semi-structured interview filled out by a geropsychologist, a psychologist expert in the psychology of aging [43]; (iii) good cognitive functioning, with a Montreal Cognitive Assessment-BLIND score ≥ 17 [44]; and, (iv) absence of depression (score ≤ 5 on the Geriatric Depression Scale-15; [45]). Out of 232 individuals contacted, eight were excluded for not meeting the inclusion criteria, resulting in a final sample of 224 community-dwelling middle-aged and older adults (age range: 46–85 years; see Table 1). All participants provided informed consent and expressed their willingness to participate in the study.

### 2.2. Materials

#### 2.2.1. Quality of Life

The World Health Organization Quality of Life-Bref (WHOQOL-BREF; [8]) is a 26-item questionnaire evaluating QoL, with two questions addressing overall QoL in the past 2 weeks and subsequent items assessing four domains: physical health (7 items; “To what extent do you feel that physical pain prevents you from doing what you need to do?”), psychological health (6 items; “How satisfied are you with yourself?”), social relationships (3 items; “How satisfied are you with your personal relationships?”), and environment (8 items; “How satisfied are you with the condition of your living place?”). Each item uses a 5-point Likert scale from 1 (not at all) to 5 (completely), with higher scores indicating better QoL. The instrument shows good reliability (α from 0.68 to 0.82). Raw scores for total score and all domains were transformed into a 0–100 scale [46], where higher scores indicate better QoL. The dependent variables were the total score, and the scores for each of the four domains (physical and psychological health, social relationships, environment).

#### 2.2.2. Personal Views of Aging

*Awareness of Age-Related Change (AARC).* The AARC (adapted from [19]) is a 50-item questionnaire comprising two scales: AARC-Gains (25 items; “With my increasing age, I realize that … I enjoy many things more intensively”) and AARC-Losses (25 items; “With my increasing age, I realize that … learning new things takes more time and effort”), with good reliability (α = 0.89 and 0.90). Each scale addresses life and behavioral domains (e.g., health/physical functioning, cognitive functioning, interpersonal relationships, sociocognitive and socioemotional functioning, lifestyle engagement) using a 5-point Likert scale from 1 (not at all) to 5 (very much). The dependent variables were the global scores for the two scales—AARC-Gains and AARC-Losses—with higher scores indicating greater awareness of age-related gains and losses, respectively.

*Felt Age (FA).* Subjective or FA was assessed with a single-item request: “Please indicate the age that you feel from 0 to 120 years.” Relative discrepancy scores were calculated as follows: subjective age − chronological age/chronological age [31], which was the dependent variable. Higher scores reflected feeling “younger” than one’s chronological age.

#### 2.2.3. Psychological Resilience

The Connor–Davidson Resilience Scale (CD-RISC-10; [33]) is a questionnaire consisting of 10 items (e.g., “Able to adapt to change”) used to assess psychological resilience. It uses a 5-point Likert scale from 0 (not true at all) to 4 (true nearly all the time) and demonstrates good reliability (α = 0.80). The dependent variable was the total score, with higher scores indicate greater resilience.

### 2.3. Procedure

After giving their written informed consent, all participants attended a 90 min individual session remotely (via Zoom or Skype) with a trained experimenter so that they could complete a series of tasks and questionnaires including, in the following order, a semi-structured interview on sociodemographic information (e.g., age, gender, and years of education) and health status (presence of serious health issues and/or use of medication such as antidepressants and/or anxiolytics), FA, MoCA-BLIND, WHOQOL-BREF, AARC, CD-RISC-10, and GDS-15. Other measures were administered and analyzed for different purposes [42].

### 2.4. Statistical Analysis

First, we conducted Pearson’s correlations between all variables of interest (see Supplementary Material). To examine the variance explained in overall QoL and its domains (i.e., physical and psychological health, social relationships, environment), multiple linear regression models were run (Table 2 and Table 3) with QoL and its domains as dependent variables, personal VoA (i.e., FA, AARC-Gains, and AARC-Losses) and psychological resilience as the independent variables (i.e., predictors), and sociodemographic factors (i.e., chronological age, gender, and education) as the control variables.

Then, to simultaneously explore the associations between personal VoA, psychological resilience, and QoL, we conducted structural equation modeling (SEM; path analyses).

Path analyses included personal VoA (FA, AARC-Gains, and AARC-Losses) as independent variables, psychological resilience as the mediating variable, and QoL (overall and by domains) as the dependent variable. More specifically, we tested the following relationships: (a) the direct effect of personal VoA on QoL, consistent with their impact on health-related outcomes of QoL (e.g., [4]); (b) the direct effect of psychological resilience on QoL, reflecting its established role in well-being and aging (e.g., [23]); and (c) the mediating role of psychological resilience in the relationship between personal VoA and QoL [26]. Because FA and AARC represent related but distinct aspects of personal VoA (see [19]) and may operate through different pathways (see [1]), we tested path analyses considering FA or AARC as predictors of either overall QoL or its four domains, separately (Table 4 and Table 5). In Supplementary Material, Part 2, all the path models for QoL domains are reported.

We included sociodemographic variables as covariates given their known influence on QoL. All parameters were standardized to facilitate interpretation of the results. A well-fitting SEM was defined by the following criteria: comparative fit index (CFI) > 0.95, Tucker–Lewis index (TLI) > 0.95, standardized root mean square residual (SRMR) < 0.05, and root mean square error of approximation (RMSEA) < 0.08 [47]. We conducted all analyses in R [48] using the *lavaan* package.

## 3. Results

### 3.1. Regression Analyses

#### 3.1.1. Overall QoL

The predictors explained 38% of the variance in overall QoL (Table 2). Higher education and greater psychological resilience were significantly associated with better QoL. As for personal VoA, AARC-Gains positively predicted QoL, whereas AARC-Losses showed a strong negative association with it (Figure S1). No other variables were significant.

#### 3.1.2. QoL Domains

Regression models for the QoL domains are shown in Table 3.

*Physical Health.* All predictors explained 32% of the variance. AARC-Losses were the only significant negative predictor, with greater awareness of age-related losses linked to lower physical QoL.

*Psychological Health.* All predictors explained 25% of the variance. Education was positively related to psychological health. AARC-Losses were negatively associated with psychological health, whereas psychological resilience was significantly related to better psychological health.

*Social Relationships.* All predictors explained 20% of the variance. Chronological age was a negative predictor. Regarding personal VoA, only AARC-Losses were a negative predictor of the social domain.

*Environment.* All predictors explained 16% of the variance. Older chronological age and higher education were significantly related to better environmental evaluation. FA was positively associated with environmental evaluation, whereas AARC-Losses were negatively associated with it. Psychological resilience was positively associated with the environmental evaluation.

### 3.2. Path Analyses

#### 3.2.1. FA and Psychological Resilience on Overall QoL

FA significantly and negatively predicted psychological resilience (β = −0.219, *p* = 0.001), indicating that feeling younger than one’s chronological age was associated with higher psychological resilience. In turn, psychological resilience positively predicted QoL (β = 0.311, *p* < 0.001). Although no direct, or total, effect of FA on QoL emerged, a significant indirect effect through psychological resilience was found (β = −0.068, *p* = 0.005): the relationship between feeling younger and having a better QoL operates via greater psychological resilience.

**Figure 1:**
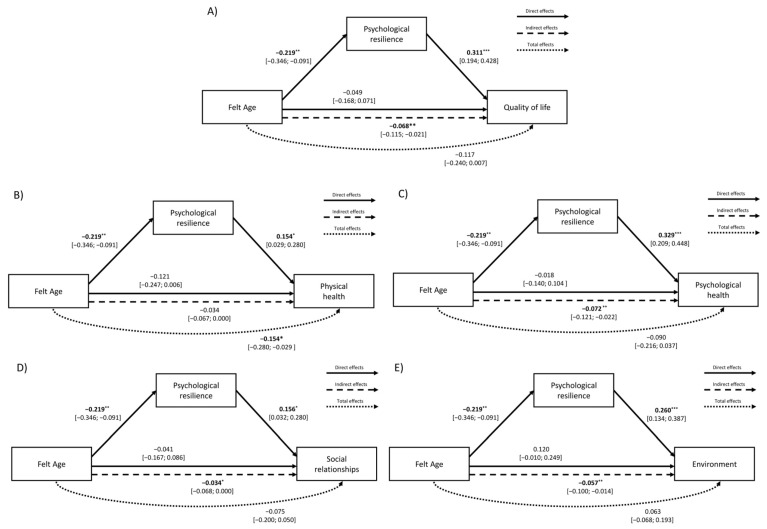
Path model showing the effects of FA (differential score) and psychological resilience on (**A**) overall QoL and its domains: (**B**) physical health, (**C**) psychological health, (**D**) social relationships, and (**E**) environment. Note. * *p* < 0.05, ** *p* < 0.01, *** *p* < 0.001.

Among the control variables, only higher education (β = 0.199, *p* = 0.002) and gender (being female) were significant predictors (β = 0.405, *p* = 0.003) of better QoL (Figure 1; Table 4).

#### 3.2.2. FA and Psychological Resilience on QoL Domains

Path model results for the QoL domains are shown in Supplementary Tables S2 and S3 and Figure 1.

*Physical Health.* FA negatively predicted psychological resilience (β = −0.219, *p* = 0.001), indicating that feeling younger than one’s chronological age is associated with higher resilience. In turn, psychological resilience was positively associated with physical health (β = 0.154, *p* = 0.016). Only the total effect of FA on physical health was significant (β = −0.154, *p* = 0.016), suggesting that feeling younger is associated with better physical health, partly through psychological resilience. Among the control variables, younger chronological age (β = −0.192, *p* = 0.005) and female gender (β = 0.329, *p* = 0.023) were associated with better physical health, while education was not.

*Psychological Health.* As in the other models (overall QoL and other domains), FA negatively predicted psychological resilience (β = −0.219, *p* = 0.001), which in turn predicted higher psychological health (β = 0.329, *p* < 0.001). Ony the indirect effect of FA via resilience was significant (β = –0.072, *p* = 0.004), suggesting that the impact of FA operates mainly through resilience. Among control variables, chronological age predicted psychological health (β = −0.149, *p* = 0.023), and education showed a positive effect (β = 0.158, *p* = 0.015) whereas gender did not (Figure 1).

*Social Relationships.* FA again predicted only higher psychological resilience (β = −0.219, *p* = 0.001), which was positively associated with social QoL (β = 0.156, *p* = 0.014). FA on social QoL was only indirect effect via resilience (β = −0.034, *p* = 0.047), indicating that feeling younger benefits social relationships through greater resilience. Considering the control variables, only chronological age was negatively associated with social relationships (β = −0.261, *p* < 0.001).

*Environment.* FA negatively predicted psychological resilience (β = −0.219, *p* = 0.001; see previous models), which was positively related to environmental QoL (β = 0.260, *p* < 0.001). Only the indirect effect through resilience was significant (β = −0.057, *p* = 0.010), suggesting a mediating role of psychological resilience on the association between feeling younger and better environment QoL.

Among control variables, only chronological age (β = 0.137, *p* = 0.049) and education (β = 0.197, *p* = 0.004) were positively associated with environmental QoL.

#### 3.2.3. AARC and Psychological Resilience on Overall QoL

Both AARC-Losses (β = −0.326, *p* < 0.001) and AARC-Gains (β = 0.392, *p* < 0.001) showed significant direct effects on psychological resilience, indicating that perceiving more age-related gains is associated with higher resilience, whereas perceiving more age-related losses is related to lower resilience. Psychological resilience, in turn, was positively associated with QoL (β = 0.156, *p* = 0.011), indicating that older adults with higher resilience experience greater QoL. Both AARC-Losses (β = −0.419, *p* < 0.001) and AARC-Gains (β = 0.138, *p* = 0.022) also directly predicted QoL. Significant indirect effects mediated by psychological resilience emerged for both AARC-Losses (β = −0.051, *p* = 0.022) and AARC-Gains (β = 0.061, *p* = 0.018), indicating that perceiving more gains or fewer losses benefits QoL through enhanced resilience. Total effects confirmed this pattern, showing a strong negative total effect of AARC-Losses on QoL (β = −0.470, *p* < 0.001) and a moderate positive total effect of AARC-Gains (β = 0.199, *p* < 0.001).

**Figure 2:**
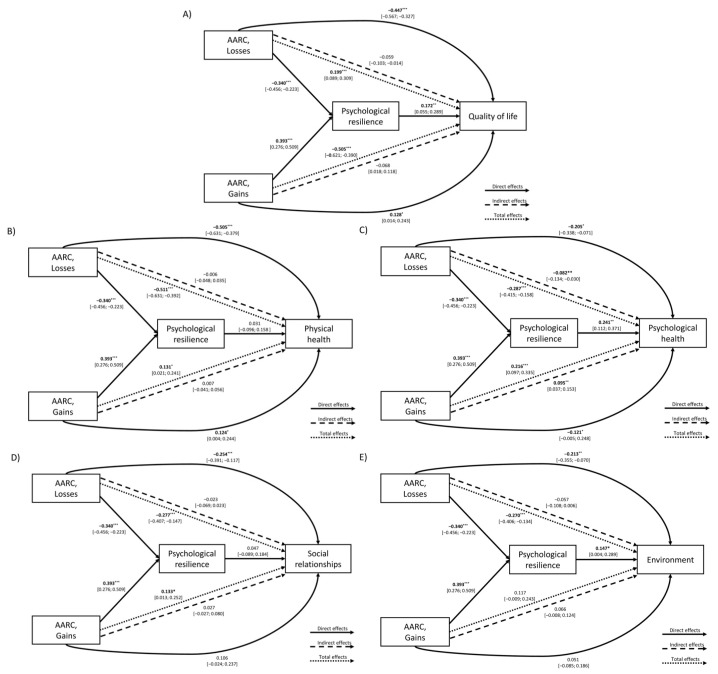
Path model showing the effect of AARC (Gains and Losses) and psychological resilience on (**A**) overall QoL and its domains: (**B**) physical health, (**C**) psychological health, (**D**) social relationships, and (**E**) environment. Note. * *p* < 0.05, ** *p* < 0.01, *** *p* < 0.001.

Among the control variables, only education and gender (being female) were positively associated with QoL (β = 0.164, *p* = 0.007; β = 0.289, *p* = 0.028, respectively) (Table 5; Figure 2).

#### 3.2.4. AARC and Psychological Resilience on QoL Domains

Path model results for the QoL domains are shown in Supplementary Tables S4 and S5 and in Figure 2.

*Physical Health.* AARC-Losses negatively predicted psychological resilience (β = −0.340, *p* < 0.001), whereas AARC-Gains positively predicted it (β = 0.393, *p* < 0.001). Only AARC-Losses and Gains (not resilience) had a significant (negative and positive, respectively) effect on physical health (AARC-Losses: β = −0.505, *p* < 0.001; AARC-Gains: β = 0.124, *p* = 0.043), suggesting that perceiving more losses relates to poorer physical functioning, whereas perceiving more gains relates to better physical health. Indirect effects through resilience were not significant for either AARC-Losses or AARC-Gains. Nonetheless, the total effects remained significant for both AARC dimensions: negative for AARC-Losses (β = −0.511, *p* < 0.001) and positive for AARC-Gains (β = 0.131, *p* = 0.019). None of the control variables (chronological age, gender, education) were significant.

*Psychological Health.* As in all other models, AARC-Losses was negatively, and AARC-Gains positively, associated with psychological resilience. Psychological resilience significantly predicted psychological health (β = 0.241, *p* < 0.001). AARC-Losses also directly and negatively related to psychological health (β = −0.205, *p* = 0.006), whereas AARC-Gains had a positive and significant direct effect (β = 0.121, *p* = 0.033). Significant indirect effects also emerged: AARC-Losses negatively affected psychological health through psychological resilience (β = −0.082, *p* = 0.002), and AARC-Gains positively via psychological resilience (β = 0.095, *p* = 0.001). Total effects were significant for both AARC-Losses (β = −0.287, *p* < 0.001) and AARC-Gains (β = 0.216, *p* < 0.001), indicating that perceptions of age-related changes (in terms of gains and/or losses) influence psychological health, partly through their influence on psychological resilience. Among the control variables, only education was positively associated with psychological health (β = 0.144, *p* = 0.012).

*Social Relationships.* Consistent with the other models, AARC-Losses were linked to lower psychological resilience and AARC-Gains to higher resilience. AARC-Losses had a significant direct negative effect on social relationships in QoL (β = −0.254, *p* < 0.001), whereas AARC-Gains did not show a significantly direct effect on it (β = 0.106, *p* = 0.110). Psychological resilience did not significantly predict social relationships, and accordingly, no indirect effects of AARC-Losses and AARC-Gains through resilience emerged. Nevertheless, total effects were significant for both AARC dimensions: negative for AARC-Losses (β = −0.277, *p* < 0.001) and positive for AARC-Gains (β = 0.133, *p* = 0.030). Among the control variables, only chronological age was negatively associated with social relationships (β = −0.198, *p* = 0.003).

*Environment.* As observed across domains, AARC-Losses predicted lower resilience and AARC-Gains higher psychological resilience. Psychological resilience significantly predicted environmental QoL (β = 0.168, *p* = 0.018). Only AARC-Losses, but not AARC-Gains, had a significant direct negative effect (β = −0.213, *p* = 0.003). Both indirect effects through psychological resilience were significant: negative for AARC-Losses (β = −0.057, *p* = 0.028) and positive for AARC-Gains (β = 0.066, *p* = 0.025). Total effects indicated that AARC-Losses had a significant overall negative impact on environmental QoL (β = −0.270, *p* < 0.001), while AARC-Gains was not significant (β = 0.117, *p* = 0.068). Among control variables, only chronological age (β = 0.162, *p* = 0.020) and education (β = 0.157, *p* = 0.023) were positively associated with environmental QoL.

## 4. Discussion

In this study, we investigated the associations between QoL (and its physical and psychological health, social relationships, and environment domains), personal VoA (in terms of FA and AARC [gains and losses]), and psychological resilience in healthy, community-dwelling middle-aged and older adults. In particular, and for the first time to our knowledge, our study examined not only the impact of FA and AARC (gains and losses) on both overall and domain-specific QoL, but also the mediating effects of psychological resilience, as a psychological pathway/factor, on these associations.

Our results, in line with our hypotheses, showed that AARC was significantly associated with overall QoL, with AARC-Gains predicting better QoL and AARC-Losses poorer QoL. These findings confirmed previous evidence linking AARC-Gains to better health outcomes related to QoL (e.g., self-rated health, well-being) and AARC-Losses to poorer outcomes [3,4]. In line with the conceptualization of AARC as a multidimensional personal VoA construct [19,49], greater AARC-Gains seem to foster an optimistic outlook and focus on positive aspects of life, contributing to the improvement of perceived QoL, whereas greater AARC-Losses may reflect self-perceptions of aging-related functional declines/negative changes that negatively impact QoL. Such a pattern of results extends the influence of AARC to overall QoL.

Looking at QoL domains, differentiated patterns emerged. In line with previous evidence linking negative self-perceptions of aging to various poorer health outcomes (e.g., [4]), greater AARC-Losses consistently predicted lower QoL across all domains. In contrast, AARC-Gains did not emerge as a significant predictor for any QoL domains, when controlling for sociodemographic variables (age, gender, education) and resilience. This result is consistent with cross-sectional evidence (e.g., [19]) showing that AARC-Losses are more strongly associated with poorer functional health and well-being than AARC-Gains (see [4] for a meta-analysis). Overall, this pattern of findings suggests that awareness of age-related losses have a stronger impact on QoL than being aware of age-related gains, even in healthy middle-aged and older adults.

By contrast, FA was not significantly related to overall QoL. This lack of association may reflect our healthy, homogeneous sample and the QoL measure used (WHOQOL-BREF), which might have reduced variability. Previous studies linking FA to QoL typically involved clinical or more vulnerable populations [21,50], and focused on narrower outcomes (e.g., mortality, physical functioning [36,51,52,53]), which could explain this discrepancy.

Interestingly, a significant, though small, association emerged only in the QoL environment domain, where feeling older than one’s chronological age was related to better perceived QoL in the physical living environment. This might suggest a stronger place attachment or environmental appreciation among those who feel older, aligning with previous findings of satisfaction/attachment with one’s surroundings with advancing age (e.g., [54]). FA, being a unidimensional VoA construct (e.g., [31]), may thus influence specific QoL domains—particularly those related to personal perceptions of daily living contexts (i.e., QoL in environment)—more than on other domains directly associated with physical or psychological health or social functioning. The present findings are consistent with conceptual models suggesting that FA often affects health and developmental outcomes—and, by extension, QoL—indirectly through mediating psychological mechanisms (e.g., [55,56,57]), such as psychological resilience, as confirmed by our path models (see below).

As expected, psychological resilience, in line with our hypothesis and previous evidence [25,26], emerged as a significant predictor of better overall QoL. As an added result, it became apparent that it was related to specific domains of QoL, extending previous evidence [12,23] to aging. When examining QoL across its domains, psychological resilience was in fact positively associated with QoL in psychological health and environment domains. This association is likely explained by internal resources such as self-efficacy and mastery of the environment [58], which are known to support well-being and the perception of control and satisfaction with one’s living context. Conversely, psychological resilience was not significantly associated with QoL in physical health or social relationships. Such a pattern of findings may also reflect our participants’ generally good physical condition and stable social context, limiting the “activation” of resilience resources [12,40], which have been shown to become more relevant under specific situations (i.e., age-related physical challenges [22,59] or reduced social support [60,61]).

Furthermore, our regression models showed that chronological age *per se* does not directly affect overall QoL in adulthood and older age, a result in line with previous studies on QoL [11]. However, older participants reported poorer QoL in the social relationship domain, possibly due to reduced networks commonly reported with aging (e.g., [62]), and better QoL in terms of perceived physical living environment, perhaps reflecting greater stability, routine, or appreciation of one’s surroundings with aging (e.g., [54]). Education positively predicted both overall QoL, psychological health, and environment domains, in line with findings highlighting its role in enhancing coping and adaptation in aging [41,63]. Gender showed no significant associations with QoL, though our sample predominantly involved females.

Path models provide further insights into the relationship between VoA, psychological resilience, and QoL. In line with our hypothesis, psychological resilience was shown to be a psychological pathway linking VoA and QoL in its multidimensionality. Resilience fully mediated the relationship between a youthful FA and better overall QoL, suggesting that feeling younger than one’s chronological age may activate typical resilience resources, such as coping and adaptation, which are essential for dealing with age-related changes in daily life and supporting QoL in aging (e.g., [12,64]). At the level of QoL domains, psychological resilience also fully mediated the association between a youthful FA and better QoL in psychological health, social relationships, and environment. This pattern aligns with previous evidence highlighting resilience as a protective factor against poorer psychological health outcomes (e.g., [25,26]), and it extends these findings to QoL domains of social relationships and environment, for example, through promoting social participation and engagement in recreational, family, and community activities and facilities (e.g., [65]). Unexpectedly, FA showed no direct or indirect effect on the physical health QoL domain, in contrast to previous studies (e.g., [49,50]). Again, this reflects individual characteristics of our sample (healthy individuals without chronic health conditions). To note, the total FA effect in this QoL domain remained significant, suggesting its relevance for physical functioning through mechanisms beyond psychological resilience, a result that deserves further investigation.

Considering the pathways from AARC to QoL, both AARC-Gains and AARC-Losses influenced overall QoL both directly, confirming our regression results and in line with previous studies [4,19], as well as indirectly via psychological resilience. This suggests that the psychological pathway through which AARC influences QoL among middle-aged and older adults could lie in the fact that greater self-reflections and conscious awareness of aging-related gains or losses could heighten or buffer, respectively, the perceived availability of strengths/resources to successfully cope with life adversities/changes, thereby supporting or impacting perceived QoL.

When analyzing QoL domains, AARC-Losses had a direct impact on all QoL domains, but indirect effects through psychological resilience emerged only for psychological health and perceived physical living environment. Conversely, AARC-Gains had a direct impact on QoL in physical and psychological health, but not on social relationships and environment, while showing an indirect effect on psychological health and perceived physical living environment through psychological resilience. From a psychological perspective, individuals more aware of aging-related losses may face challenges with reduced adaptive resources, negatively affecting their psychological health and satisfaction with their living environment. In contrast, those aware of aging-related gains exhibited higher psychological resilience, drawing on self-efficacy and sense of control, which may foster more constructive adaptation and, in turn, better psychological and environment aspects of QoL. Overall, the total effects were consistently stronger for AARC-Losses than for AARC-Gains across all QoL domains, in line with previous evidence that age-related losses exert greater impact on well-being than gains [4,20], here extended to overall QoL and its domains.

Despite the interesting results, some limitations should be acknowledged. First, the cross-sectional design of our study does not allow for causal or bidirectional conclusions. Second, the sample included healthy, community-dwelling older adults, with a high proportion of women, which may limit the generalizability of results to a broader population.

Although our sample covers both midlife and older adulthood and thus provides insight into subjective aging across this broad age range, the sample size and cross-sectional design did not allow for a reliable examination of subgroup effects in terms of differences between middle-aged and older adults. Future longitudinal studies should therefore replicate and extend these findings using larger and more heterogeneous samples (both in terms of sociodemographic and health-related characteristics) and consider other influencing variables (socioeconomic status) (e.g., [66]) and VoA measures [67] to fully analyze the interplay among personal VoA, psychological resilience, and QoL over time. Moreover, other relevant individual factors (e.g., living [physical and social] conditions and personality traits) were not assessed; thus, future research could account for these influences because they may affect both VoA and QoL. Nonetheless, the merit of this study is in shedding (some) light on how subjective experiences of aging influence QoL in midlife and older age.

## 5. Conclusions

In conclusion, our findings emphasize and confirm the complex interplay between global (FA) and multidimensional (AARC) personal VoA and QoL, in its multidimensionality, highlighting the importance of regarding the multifaceted nature of both constructs to better capture their associations.

They also provide, for the first time, cross-sectional evidence of resilience as a “psychological pathway” explaining how personal VoA can influence various aspects of QoL in midlife and older age, depending on the VoA facet and QoL dimension considered. They thus contribute to identifying factors that favor and sustain QoL in older age. Therefore, assessing personal VoA alongside psychological resources such as resilience could help identify individuals at risk of holding maladaptive, ageist self-views, providing modifiable risk factors and valuable targets for interventions to sustain QoL and healthy, active aging.

## Figures and Tables

**Table 1:** Descriptive statistics (mean and standard deviations) of sociodemographic and all measures of interest.

	M	SD
Chronological age (years)	61.54	9.87
Education (years)	11.87	3.86
Gender (%)	75	-
MOCA-BLIND	19.41	1.54
GDS−15	1.92	1.57
Personal VoA		
FA	−0.13	0.13
AARC-Gains	84.81	15.19
AARC-Losses	54.22	14.71
Resilience		
CD-RISC-10	35.90	6.23
QoL		
WHOQOL-BREF, Total score	96.33	9.75
WHOQOL-BREF, Physical health	72.83	13.28
WHOQOL-BREF, Psychological health	59.65	10.61
WHOQOL-BREF, Social relationships	67.67	14.46
WHOQOL-BREF, Environment	64.01	10.77

Note. FA = felt age; AARC = Awareness of Age-Related Change-50; CD-RISC-10 = Connor–Davidson Resilience Scale; WHOQOL-BREF = World Health Organization Quality of Life questionnaire Bref-version.

**Table 2:** Linear regression model (standardized betas, CI, and *p* values) for overall QoL.

	β	95% CI	*p*
Chronological Age	−0.009	[−0.128; 0.111]	0.889
Gender	0.107	[−0.033; 4.872]	0.053
Education	0.151	[0.084; 0.677]	**0.012**
FA	0.025	[−6.630; 1.358]	0.666
AARC-Gains	0.131	[0.008; 0.159]	**0.030**
AARC-Losses	−0.452	[−0.382; −0.217]	**<0.001**
CD-RISC-10	0.176	[0.085; 0.465]	**0.005**
R^2^/R^2^ adjusted	0.364/0.384		

Note. FA = felt age; AARC = Awareness of Age-Related Change-50; CD-RISC-10 = Connor–Davidson Resilience Scale; WHOQOL-BREF = World Health Organization Quality of Life questionnaire Bref-version. In bold type *p* values ≤ 0.01.

**Table 3:** Linear regression model (standardized betas, CI, and *p* values) for QoL domains.

	WHOQOL-BREF, Physical Health			WHOQOL-BREF, Psychological Heath			WHOQOL-BREF, Social Relationships			WHOQOL-BREF, Environment		
	β	*95% CI*	*p*	β	*95% CI*	*p*	β	*95% CI*	*p*	β	*95% CI*	*p*
Chronological Age	−0.064	[−0.257; 0.084]	0.319	−0.099	[−0.250; 0.038	0.147	−0.198	[−0.492; −0.088]	**0** **.005**	0.199	[0.063; 0.371]	**0** **.006**
Gender	0.066	[−1.487; 5.525]	0.258	0.078	[−1.032; 4.878	0.201	0.082	[−1.396; 6.916]	0.192	0.018	[−2.738; 3.613]	0.786
Education	0.068	[−0.191; 0.658]	0.279	0.145	[0.041; 0.756]	**0** **.029**	0.081	[−0.200; 0.806]	0.237	0.170	[0.091; 0.859]	**0** **.016**
FA	−0.041	[−16.385; 7.903]	0.492	0.019	[−8.704; 11.762	0.769	0.002	[−14.179; 14.609]	0.977	0.159	[2.326; 24.323]	**0** **.018**
AARC-Gains	0.120	[−0.003; 0.213]	0.056	0.123	[−0.005; 0.177]	0.064	0.106	[−0.027; 0.229]	0.121	0.065	[−0.052; 0.144]	0.356
AARC-Losses	−0.497	[−0.566; −0.331]	**<0.001**	−0.208	[−0.249; −0.051]	**0** **.003**	−0.254	[−0.390; −0.111]	**<0.001**	−0.244	[−0.285; −0.072]	**0** **.001**
CD-RISC-10	0.013	[−0.244; 00.300]	0.839	0.244	[0.186; 0.644]	**<0.001**	0.068	[−0.165; 0.480]	0.337	0.189	[0.081; 0.574]	**0** **.009**
R^2^/R^2^ adjusted	0.324/0.295			0.245/0.220			0.197/0.171			0.155/0.127		

Note. FA = felt age; AARC = Awareness of Age-Related Change−50; CD-RISC-10 = Connor–Davidson Resilience Scale; WHOQOL-BREF = World Health Organization Quality of Life-BREF. In bold type *p* values ≤ 0.01.

**Table 4:** Standardized direct, indirect, and total effects on WHOQOL-BREF, total score from the structural equation model.

Model Pathways	β [95% CI]	SE	*z*	*p*
Direct effect				
a1 * FA→CD-RISC-10	−0.219 [−0.346; −0.091]	0.065	−3.354	**0.001**
b1 * FA→WHOQOL-BREF, tot	−0.049 [−0.168; 0.071]	0.061	−0.798	0.425
b2 * CD-RISC-10→WHOQOL-BREF, tot	0.311 [0.194; 0.428]	0.060	5.205	**<** **0.001**
Age→WHOQOL-BREF, tot	−0.123 [−0.250; 0.003]	0.064	−1.917	0.055
Education→WHOQOL-BREF, tot	0.199 [0.074; 0.324]	0.064	3.125	**0.002**
Gender→WHOQOL-BREF, tot	0.405 [0.138; 0.671]	0.136	2.975	**0.003**
Indirect effect				
a1 * b2 FA→CD-RISC-10→WHOQOL-BREF, tot	−0.068 [−0.115; −0.021]	0.024	−2.819	**0.005**
Total effect				
b1 + (a1 * b2)	−0.117 [−0.240; 0.007]	0.063	−1.854	0.064

Note. Standardized estimates are reported with 95% confidence intervals (CI), standard errors (SE), *z* values, and *p* values. FA = felt age; AARC = Awareness of Age-Related Change-50; CD-RISC-10 = Connor–Davidson Resilience Scale; WHOQOL-BREF = World Health Organization Quality of Life-BREF. In bold type *p* values ≤ 0.01.

**Table 5:** Standardized direct, indirect, and total effects on WHOQOL-BREF, total from the structural equation model.

Model Pathways	β [95% CI]	SE	*z*	*p*
Direct effect				
a1 * AARC-Losses→CD-RISC-10	−0.340 [−0.456; −0.223]	0.059	−5.715	**<0.001**
a2 * AARC-Gains→CD-RISC-10	0.393 [0.276; 0.509]	0.059	6.605	**<0.001**
b1 * AARC-Losses→WHOQOL-BREF, tot	−0.447 [−0.567; −0.327]	0.061	−7.284	**<0.001**
b2 * AARC-Gains→WHOQOL-BREF, tot	0.128 [0.014; 0.243]	0.058	2.205	**0.027**
b3 * CD-RISC-10→WHOQOL-BREF, tot	0.172 [0.055; 0.289]	0.060	2.888	**0.004**
Age→WHOQOL-BREF, tot	−0.014 [−0.129; 0.101]	0.059	−0.244	0.807
Education→WHOQOL-BREF, tot	0.149 [0.035; 0.263]	0.058	2.558	**0.011**
Gender→WHOQOL-BREF, tot	0.246 [0.001; 0.491]	0.125	1.969	**0.049**
Indirect effect				
a1 * b3 AARC-Losses→CD-RISC-10→WHOQOL-BREF, tot	−0.059 [−0.103; −0.014]	0.023	−2.577	**0.010**
a2 * b3 AARC-Gains→CD-RISC-10→WHOQOL-BREF, tot	0.068 [0.018; 0.118]	0.026	2.646	**0.008**
Total effect				
b1 + (a1 * b3)	−0.505 [−0.621; −0.390]	0.059	−8.596	**<0.001**
b2 + (a2 * b3)	0.196 [0.090; 0.302]	0.054	3.612	**<0.001**

Note. Standardized estimates are reported with 95% confidence intervals (CI), standard errors (SE), *z* values, and *p* values. AARC = Awareness of Age-Related Change-50; CD-RISC-10 = Connor–Davidson Resilience Scale; WHOQOL-BREF = World Health Organization Quality of Life-BREF. In bold type *p* values ≤ 0.01.

## Data Availability

The data that support the findings of this study are available on request from the corresponding author. The data are not publicly available due to the confidential nature of the dataset collected.

## Supplementary Materials

The following supporting information can be downloaded at: https://www.mdpi.com/article/10.3390/healthcare13222906/s1, Table S1: Correlations between measures of interest; Table S2: Model fit indices for path models examining the relationships between felt age, psychological resilience, and QoL (overall and its domains); Table S3: Standardized direct, indirect, and total effects of felt age and psychological resilience on WHOQOL-BREF domains (physical and psychological health, social relationships, and environment); Table S4: Model fit indices for path models examining the relationships between AARC (gains and losses), psychological resilience, and QoL (overall and its domains); Table S5: Standardized direct, indirect, and total effects of AARC (gains and losses) and psychological resilience on WHOQOL-BREF domains (physical and psychological health, social relationships, and environment); Figure S1: Overall QoL as a function of personal VoA measures (AARC-Losses, AARC-Gains, and felt age); Figure S2: QoL domains as a function of personal VoA measures (AARC-Losses, AARC-Gains, and felt age).
