# Supplementary material for: Associations Between Personal Views of Aging and Quality of Life in Midlife and Older Age: The Mediating Role of Psychological Resilience
Source: Healthcare (Basel). 2025 Nov 14;13(22):2906. doi: 10.3390/healthcare13222906 (PMC12652676; doi:10.3390/healthcare13222906)

Associations Between Personal Views of Aging and Quality of Life in Midlife and Older Age: The Mediating Role of Psychological Resilience

SUPPLEMENTARY MATERIAL

Summary of the contents

PART 1 – Results from correlation analyses
PART 2 – Path models for QoL domains
PART 3 – Figures

PART 1 – RESULTS FROM CORRELATION ANALYSES

Personal VoA and QoL

There was a significant large negative correlation between the QoL overall score and AARC-Losses ($r = –.542$, $p <.001$), suggesting that greater awareness of age-related losses is associated with a lower perception of QoL. However, no significant correlation was observed between the QoL overall score and AARC-Gains. Similarly, no significant association was found between the QoL overall score and felt age.

Regarding QoL domains, there was a significant negative correlation between AARC-Losses and the physical ($r = –.541$, $p <.001$), psychological ($r = –.340$, $p <.001$), and social ($r = –.355$, $p <.001$) domains of QoL. This pattern suggests that perceiving age-related losses is associated with a lower perception of QoL in psychological and physical health, as well as in social relationships and environmental perceptions.

AARC-Gains was not significantly associated with any QoL domain, indicating that perceiving age-related gains is not related to perceived QoL.

Felt age was negatively associated only with the physical domain ($r = –.146$, $p = .029$), suggesting that feeling older is associated with poorer physical health functioning.

Psychological resilience and QoL

Psychological resilience was strongly and positively correlated with the QoL overall score ($r = .369$, $p <.001$), indicating that greater resilience is associated with a better perceived quality of life. Resilience was also significantly and positively correlated with all QoL domains: physical ($r = .219$, $p <.001$), psychological ($r = .369$, p <.001), social (r = .202, $p = .002$), and environmental ($r = .256$, $p <.001$). These results suggest that higher psychological resilience is linked to better functioning across multiple dimensions of QoL.

Personal VoA and Psychological resilience

There was a significant negative correlation between AARC-Losses and psychological resilience ($r = –.278$, $p <.001$), indicating that perceiving more age-related losses is associated with lower resilience. AARC-Gains showed no significant association with resilience.

Felt age was negatively associated with resilience ($r = –.219$, $p <.001$), suggesting that feeling older than one’s chronological age may be linked to reduced psychological resilience.

Sociodemographic variables

Age was negatively correlated with the QoL overall score ($r = –.199$, $p = .003$), as well as with the physical ($r = –.222$, $p < .001$), psychological ($r = –.218$, $p < .001$), and social ($r = –.296$, $p < .001$) domains, but not with the environmental domain. Age was also positively associated with AARC-Losses ($r = .304$, $p < .001$), suggesting that older individuals tend to report greater awareness of age-related losses.

In addition, age was negatively associated with felt age ($r = –.147$, $p = .028$), indicating that older individuals tend to feel slightly younger than their chronological age.

Gender showed small but significant positive correlations with the QoL overall score ($r = .202$, $p = .002$) and with the physical domain ($r = .158$, $p = .018$). Gender was not significantly correlated with psychological resilience.

Education was positively correlated with the QoL overall score ($r = .284$, $p < .001$) and its domains – physical ($r = .222$, $p < .001$), psychological ($r = .254$, $p < .001$), social ($r = .223$, $p < .001$), and environmental ($r = .168$, $p = .012$) – but not with psychological resilience. Education was also negatively associated with AARC-Losses ($r = –.196$, $p < .001$), indicating that individuals with higher education perceived fewer age-related losses.

Table S1. Correlations between measures of interest.

	Age	1	2	3	4	5	6	7	8	9	10
Gender (1)	.053										
Education (2)	-.388***	-.012									
FA (3)	-.147*	-.094	-.049								
AARC-Gains (4)	.088	-.052	-.121	-.110							
AARC-Losses (5)	.304***	-.196**	-.282***	.178**	.158*						
CD-RISC 10 (6)	-.048	.103	.117	-.219***	.339***	-.278***					
WHOQOL-BREF, Total score (7)	-.199**	.202**	.284***	-.125	.092	-.542***	.369***				
WHOQOL-BREF, Physical health (8)	-.222***	.158*	.222***	-.146*	.034	-.541***	.219***	.787***			
WHOQOL-BREF, Psychological health (9)	-.218**	.129	.254***	-.085	.141*	-.340***	.369***	.730***	.441***		
WHOQOL-BREF, Social relationships (10)	-.296***	.122	.223***	-.053	.058	-.355***	.202**	.597***	.318***	.400***	
WHOQOL-BREF, Environment (11)	.033	.075	.168*	.028	.069	-.249***	.256***	.731***	.335***	.463***	.322***

Note: FA = Felt Age; AARC = Awareness of Age-Related Change-50; CD- RISC 10 = Connor-Davidson Resilience Scale; WHOQOL-BREF = World Health Organization Quality of Life questionnaire, BREF.

PART 2 – PATH MODELS FOR QoL DOMAINS

Table S2. Model fit indices for path models examining the relationships between felt age, psychological resilience, and QoL (overall and its domains).

Model (outcome)	χ^2(df)	p	RMSEA	SRMR	CFI	NNFI
WHOQOL-BREF, Total score	5.093 (2)	.165	.057	.039	.970	.910
WHOQOL-BREF, Physical health	4.865 (2)	.182	.053	.032	.960	.879
WHOQOL-BREF, Psychological health	5.093 (2)	.165	.057	.039	.966	.897
WHOQOL-BREF, Social relationships	5.093 (2)	.165	.057	.037	.953	.859
WHOQOL-BREF, Environment	3.094 (2)	.213	.049	.036	.984	.944

Note. χ^2(df) = chi-square statistic with degrees of freedom; RMSEA = Root Mean Square Error of Approximation; SRMR = Standardized Root Mean Square Residual; CFI = Comparative Fit Index; NNFI = Non-Normed Fit Index (Tucker-Lewis Index); Acceptable model fit: RMSEA ≤ .08, SRMR ≤ .08, CFI/NNFI ≥ .90.

Table S3. Standardized direct, indirect, and total effects of felt age and psychological resilience on WHOQOL-BREF domains (physical and psychological health, social relationships, and environment).

Model pathways	WHOQOL-BREF, Physical health (β [95% CI], SE, z, p)	WHOQOL-BREF, Psychological health (β [95% CI], SE, z, p)	WHOQOL-BREF, Social relationships (β [95% CI], SE, z, p)	WHOQOL-BREF, Environment (β [95% CI], SE, z, p)
**Direct effect**				
a1*Felt Age → CD-RISC10	-.219 [-.346; -.091], .065, -3.354, .001	-.219 [-.346; -.091], .065, -3.354, .001	-.219 [-.346; -.091], .065, -3.354, .001	-.219 [-.346; -.091], .065, -3.354, .001
b1*Felt Age → WHOQOL-BREF	-.121 [-.247; .006], .065, -1.867, .062	-.018 [-.140; .104], .062, -.286, .775	-.041 [-.167; .086], .065, -.631, .528	.120 [-.010; .249], .066, 1.814, .070
b2*CD-RISC10 → WHOQOL-BREF	.154 [.029; .280], .064, 2.406, .016	.329 [.209; .448], .061, 5.399, <.001	.156 [.032; .280], .063, 2.468, .014	.260 [.134; .387], .065, 4.037, <.001
Age → WHOQOL-BREF	-.192 [-.326; -.058], .068, -2.808, .005	-.149 [-.277; -.020], .066, -2.267, .023	-.261 [-.394; -.127], .068, -3.831, <.001	.137 [.000; .273], .070, 1.965, .049
Education → WHOQOL-BREF	.125 [-.008; .258], .068, 1.843, .065	.158 [.031; .286], .065, 2.437, .015	.103 [-.029; .235], .067, 1.533, .125	.197 [.062; .332], .069, 2.867, .004

Gender → WHOQOL-BREF	.329 [.046; .613], .145, 2.278, .023	.239 [-.032; .511], .139, 1.728, .084	.272 [-.010; .553], .144, 1.890, .059	.126 [-.161; .414], .147, .862, .389
**Indirect effect**				
a1*b2 Felt Age → CD-RISC10 → WHOQOL-BREF	**-.034 [-.067; .000], .017, -1.955, .051**	**-.072 [-.121; -.022], .025, -2.849, .004**	**-.034 [-.068; .000], .017, -1.988, .047**	**-.057 [-.100; -.014], .022, -2.580, .010**
**Total effect**				
b1 + (a1*b2)	-.154 [-.280; -.029], .064, -2.413, .016	-.090 [-.216; .037], .064, -1.393, .164	-.075 [-.200; .050], .064, -1.172, .241	.063 [-.068; .193], .067, .940, .347

Note. Standardized estimates are reported with 95% confidence intervals (CI), standard errors (SE), z values, and p values. AARC = Awareness of Age-Related Change-50; CD-RISC10 = Connor-Davidson Resilience Scale; WHOQOL-BREF = World Health Organization Quality of Life-BREF. Domains included as columns are: WHOQOL-BREF, Phy = Physical Health, Psy = Psychological Health, Soc = Social Relationships, and Env = Environment. In bold type, p values ≤ .01.

Table S4. Model fit indices for path models examining the relationships between AARC (gains and losses), psychological resilience, and QoL (overall and its domains).

**Model (outcome)**	**χ^2(df)**	**p**	**RMSEA**	**SRMR**	**CFI**	**NNFI**
WHOQOL-BREF, Total score	3.843 (2)	.279	.036	.020	.996	.984
WHOQOL-BREF, Physical health	3.179 (2)	.365	.016	.017	.999	.996
WHOQOL-BREF, Psychological health	3.179 (2)	.365	.016	.018	.999	.996
WHOQOL-BREF, Social relationships	3.179 (3)	.365	.016	.017	.999	.995
WHOQOL-BREF, Environment	3.179 (3)	.365	.016	.018	.999	.995

Note. χ^2(df) = chi-square statistic with degrees of freedom; RMSEA = Root Mean Square Error of Approximation; SRMR = Standardized Root Mean Square Residual; CFI = Comparative Fit Index; NNFI = Non-Normed Fit Index (Tucker-Lewis Index); Acceptable model fit: RMSEA ≤ .08, SRMR ≤ .08, CFI/NNFI ≥ .90.

Table S5. Standardized direct, indirect, and total effects of AARC (gains and losses) and psychological resilience on WHOQOL-BREF domains (physical and psychological health, social relationships, and environment).

**Model pathways**	**WHOQOL-BREF, Physical health**	**WHOQOL-BREF, Psychological health**	**WHOQOL-BREF, Social relationships**	**WHOQOL-BREF, Environment**

	(β [95% CI], SE, z, p)	(β [95% CI], SE, z, p)	(β [95% CI], SE, z, p)	(β [95% CI], SE, z, p)
**Direct effects**				
a1 * AARC, Losses → CD-RISC10	**-.340 [-.456; -.223], .059, -5.715, <.001**	**-.340 [-.456; -.223], .059, -5.715, <.001**	**-.340 [-.456; -.223], .059, -5.715, <.001**	**-.340 [-.456; -.223], .059, -5.715, <.001**
a2 * AARC, Gains → CD-RISC10	**.393 [.276; .509], .059, 6.605, <.001**	**.393 [.276; .509], .059, 6.605, <.001**	**.393 [.276; .509], .059, 6.605, <.001**	**.393 [.276; .509], .059, 6.605, <.001**
b1 * AARC, Losses → WHOQOL-BREF	-.505 [-.631; -.379], .064, -7.838, <.001	-.205 [-.338; -.071], .068, -3.011, .006	-.254 [-.391; -.117], .070, -3.627, <.001	-.213 [-.355; -.070], .073, -2.923, .003
b2 * AARC, Gains → WHOQOL-BREF	.124 [.004; .244], .061, 2.024, .043	.121 [-.005; .248], .064, 1.881, .033	.106 [-.024; .237], .066, 1.597, .110	.051 [-.085; .186], .069, .737, .461
b3 * CD-RISC10 → WHOQOL-BREF	.019 [-.104; .244], .063, .297, .767	.241 [.112; .371], .066, 3.651, .001	.067 [-.066; .201], .068, .990, .322	.168 [.029; .186], .071, 2.375, .018
Age → WHOQOL-BREF	-.055 [-.175; .066], .062, -.887, .375	-.103 [-.230; .024], .065, -1.587, .204	-.198 [-.329; -.067], .067, -2.957, .003	.162 [.025; .298], .070, 2.321, .020
Education → WHOQOL-BREF	.071 [-.048; .191], .061, 1.167, .243	.144 [.018; .270], .064, 2.232, .012	.081 [-.049; .211], .066, 1.215, .224	.157 [.022; .293], .069, 2.282, .023
Gender → WHOQOL-BREF	.155 [-.102; .413], .131, 1.183, .237	.180 [-.092; .451], .138, 1.299, .204	.191 [-.089; .470], .143, 1.336, .182	.028 [-.263; .318], .148, .186, .852
**Indirect effects**				
a1 * b3 AARC, Losses → CD-RISC10 → WHOQOL-BREF	**-.006 [-.048; .035], .021, -.297, .767**	**-.082 [-.134; -.030], .027, -3.076, .002**	-.023 [-.069; .023], .023, -.976, .329	**-.057 [-.108; -.006], .026, -2.193, .028**
a2 * b3 AARC, Gains → CD-RISC10 → WHOQOL-BREF	**.007 [-.041; .056], .025, .297, .767**	**.095 [.037; .153], .030, 3.195, .001**	.027 [-.027; .080], .027, .980, .327	**.066 [.008; .124], .030, 2.235, .025**
**Total effects**				
b1 + (a1 * b3)	**-.511 [-.631; -.392], .061, -8.407, <.001**	**-.287 [-.415; -.158], .066, -4.361, <.001**	**-.277 [-.407; -.147], .066, -4.183, <.001**	**-.270 [-.406; -.134], .069, -3.888, <.001**
b2 + (a2 * b3)	**.131 [.021; .241], .056, 2.341, .019**	**.216 [.097; .335], .061, 3.556, <.001**	**.133 [.013; .252], .061, 2.175, .030**	.117 [-.009; .243], .064, 1.826, .068

PART 3 – FIGURES

Regression analyses

Figure S1. Overall QoL as a function of personal VoA measures (AARC-Losses, AARC-Gains, and Felt age).

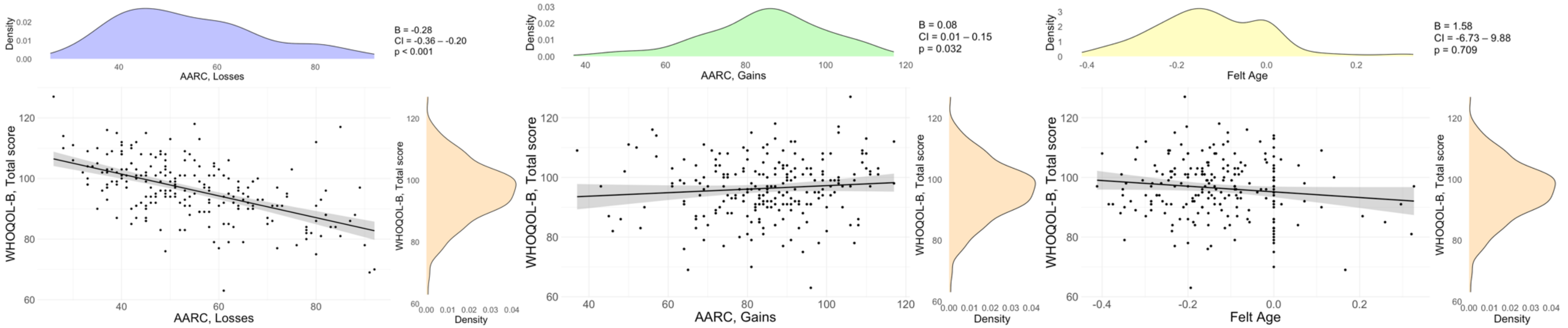

Figure S2. QoL domains as a function of personal VoA measures (AARC-Losses, AARC-Gains, and Felt age).

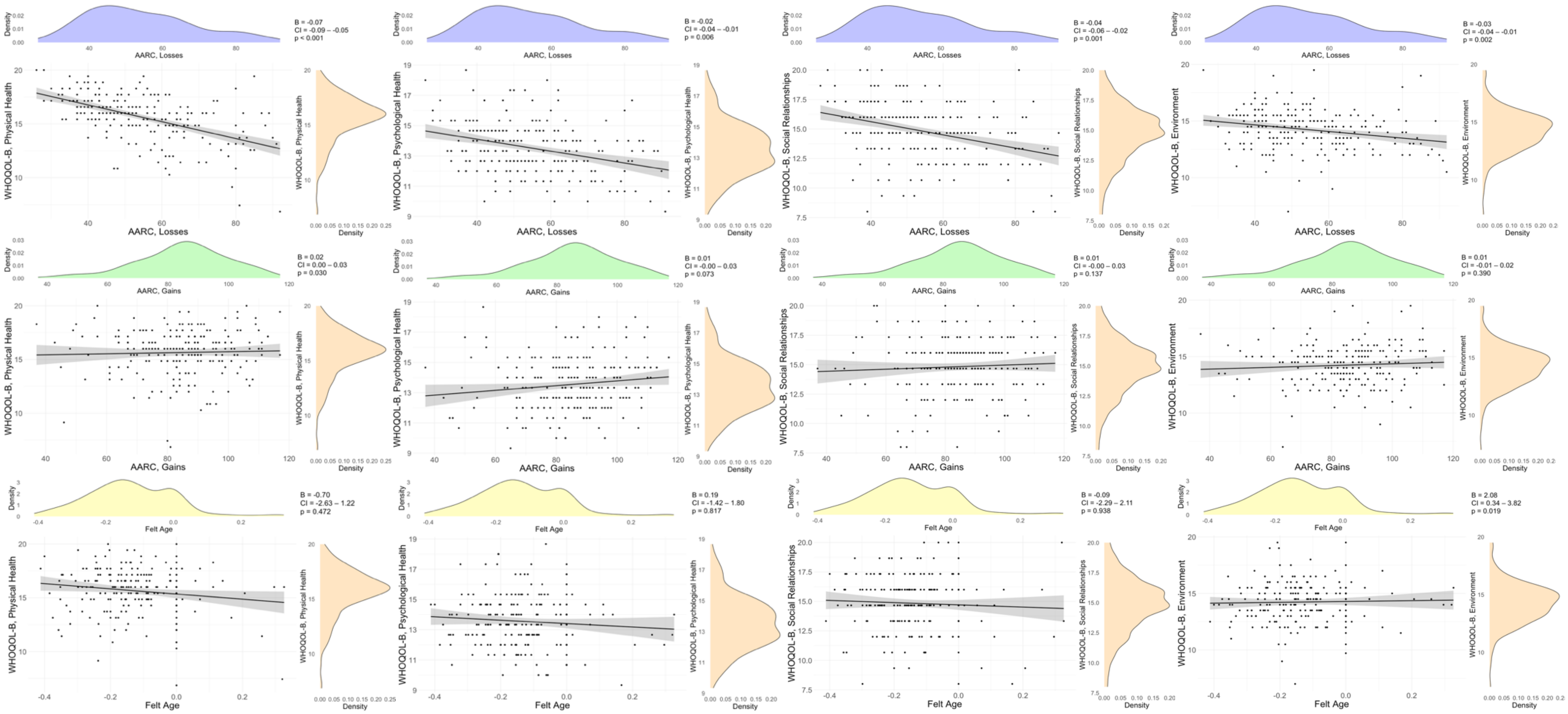

Supplement: Supplementary file 1 [file healthcare-13-02906-s001.zip › healthcare-3947271-supplementary.pdf]
